# Supplementary material for: Ethnicity/caste and child anthropometric outcomes in India using the National Family Heath Survey 2015–16 and 2019–21
Source: PLoS One. 2024 Dec 10;19(12):e0311092. doi: 10.1371/journal.pone.0311092 (PMC11630627; doi:10.1371/journal.pone.0311092)
Supplement: S1 Table — (DOCX) [file pone.0311092.s001.docx]

**Supporting Document**

**Ethnicity/Caste and Child Anthropometric Outcomes in India using the National Family Heath Survey 2015-16 and 2019-21**

**S1 Table** **Variance Inflation Factor (VIF) and Tolerance Values for Predictor Variables used in the study**

|  | **VIF** | **Tolerance** |
| --- | --- | --- |
| **Age (in months)** |  |  |
| 6-11 months | Reference |  |
| 12-17 months | 1.72 | 0.579801 |
| 18-23 months | 1.7 | 0.587887 |
| 24-35 months | 2 | 0.499461 |
| 36-47 months | 1.9 | 0.526833 |
| 48-59 months | 1.79 | 0.55837 |
| **Sex of Child** |  |  |
| Male | Reference |  |
| Female | 1.01 | 0.994134 |
| **Birth Order** |  |  |
| 1^st^ | Reference |  |
| 2^nd^ | 1.36 | 0.735997 |
| 3^rd^ or more | 1.64 | 0.608884 |
| **Normal birthweight (2.5 kgs or more)** | 1.01 | 0.9878 |
| **4 or more ANC Visits of Mother** | 1.12 | 0.896404 |
| **BMI of Mother** |  |  |
| Underweight (BMI<18.5) | Reference |  |
| Normal (BMI≥18.5 & <24.5) | 1.51 | 0.663988 |
| Overweight & Obese (BMI≥25) | 1.66 | 0.604097 |
| **Education of Mother** |  |  |
| No Formal Education | Reference |  |
| Primary Education | 1.46 | 0.686983 |
| Secondary Education | 2.2 | 0.455146 |
| Higher Education | 2.09 | 0.479363 |
| **Area of the Household** |  |  |
| Rural | Reference |  |
| Urban | 1.32 | 0.755762 |
| **Households with Women as Head** | 1 | 0.996596 |
| **Wealth Quintile** |  |  |
| Poorest | Reference |  |
| Poorer | 1.8 | 0.555669 |
| Middle | 2.04 | 0.490401 |
| Richer | 2.35 | 0.425952 |
| Richest | 2.9 | 0.345413 |
| **Ethnic Group** |  |  |
| General | Reference |  |
| Other Backward Castes | 1.85 | 0.541054 |
| Scheduled Castes | 1.73 | 0.579692 |
| Scheduled Tribes | 2.13 | 0.470406 |
| **Religion** |  |  |
| Hindu | Reference |  |
| Muslim | 1.11 | 0.90312 |
| Christian | 1.07 | 0.938899 |
| Sikh | 1.39 | 0.717672 |
| Other | 1.06 | 0.947838 |
