# Supplementary material for: Ethnicity/caste and child anthropometric outcomes in India using the National Family Heath Survey 2015–16 and 2019–21
Source: PLoS One. 2024 Dec 10;19(12):e0311092. doi: 10.1371/journal.pone.0311092 (PMC11630627; doi:10.1371/journal.pone.0311092)
Supplement: Attachment — Submitted filename: MyComments.docx [file pone.0311092.s003.docx]

My Comments on

PONE-D-24-16005

**Understanding the impact of Ethnicity/Caste and Child Anthropometric Outcomes in**

**India using the National Family Heath Survey 2015-16 and 2019-21**

Since the table numbers do not match with the referred table numbers, I would suggest the authors resubmit the paper. The following points should be taken into account before resubmitting.

1. The description of variables is too big. Instead, it would be nice if the list of variables taken in the analysis is presented in the form of a table with columns as (i) name of the variable, (ii) a brief description of the variable and (iii) the categories if it is a categorical variable. The required explanations may be given as brief as possible.
2. It is wrong to say that the “logistic model fails to account for the interaction between ethnicity and other covariates”. However, the ESTER and Blinder Oaxaca model models are welcome.
3. Table numbers and referred table numbers do not match. In fact, table numbering is not OK. After Table 1 it jumps to Table 11.
4. Table 12 is not necessary.
5. While or before carrying out the logistic regression it is necessary to test for multicollinearity of the explanatory variables.
6. Because the paper is too lengthy, I would suggest removing the figures completely.
